# Comparison of primary endpoints between publications, registries, and protocols of phase III cancer clinical trials

**DOI:** 10.18632/oncotarget.21459

**Published:** 2017-10-03

**Authors:** Fei Liang, Xinmei Guo, Sheng Zhang, Hongxi Xue, Qiang Chen, Xichun Hu

**Affiliations:** ^1^ Shanghai Cancer Center and Shanghai Medical College, Fudan University, Shanghai, China; ^2^ Interventional treatment unit, Shandong Chest Hospital, Jinan, China; ^3^ Rizhao City Hospital of Traditional Chinese Medicine, Rizhao, China; ^4^ Department of Clinical Biochemistry, School of Public Health Taishan Medical University, Taian, China

**Keywords:** primary endpoints, protocols, randomized controlled trials, cancer

## Abstract

**Background:**

Decisions by leading journals to require trial registration and to make protocols of phase III randomized clinical trials (RCTs) publicly accessible were landmark events in clinical trial reporting.

**Materials and Methods:**

We identified phase III cancer RCTs published between 2013 and 2015 in New England Journal of Medicine (*NEJM*), The Lancet, The lancet Oncology, *JAMA* and Journal of Clinical Oncology (JCO).

**Results:**

We identified 345 reports of phase III RCTs of which 217 (62.9%) had available protocols. The availability rates for *NEJM*, The Lancet, The Lancet Oncology, *JAMA* and JCO were 98.0%, 33.3%, 22.7%, 55.6% and 88.3%, respectively. Journal and publication year were significantly associated with protocol availability. Eight of 215 trials (3.7%) with English language protocols had a discrepancy in primary endpoints between publication and protocol. Discrepancies of primary endpoints between protocol and registration existed in 16 (7.7%) of 209 trials.

**Conclusions:**

The policy of providing protocols with articles reporting RCTs has not been enforced rigorously. Selective reporting of primary endpoints only remains in a small fraction of phase III trials. Further improvement in consistency between primary endpoints registered and that in protocol is necessary.

## INTRODUCTION

Selective outcome reporting, where the primary endpoint is changed, degraded or omitted, or a new one introduced, may invalidate the results of phase III randomized controlled trials (RCTs), which are regarded as the gold standard for evaluating medical interventions [1–5]. Selective reporting will hamper reproducibility of research, bias meta-analysis and result in dissemination of potentially misleading scientific results, particularly if changes were made to yield apparently positive results when analysis using the original outcome did not [2, 3, 5]. Selective reporting of the primary endpoint has been observed in as many as 62% of published trials [2].

Prompted by such concerns, the CONSORT statement was developed in 1996 by the International Committee of Medical Journal Editors to ensure clarity and transparency for the reporting of clinical trials [6]. More recently, the requirement for trial registration before participant enrollment was initiated as a precondition for publication of the trial’s findings in member journals [7, 8]. However, comparison of primary endpoints of registered RCTs with their subsequent publication indicated that selective outcome reporting was still prevalent in a substantial proportion of trials [3, 5].

Between 2009 and 2011, leading medical journals began requiring the disclosure of protocols as a criterion for publication of results of RCTs [9–11]. Public availability of protocols should reduce the frequency of selective outcome reporting, because readers and reviewers can verify that outcome measures are identical to those in the protocol. Discrepancies in primary endpoints between protocols, registries, and publications have been reported for several trials and leads to concern about possible bias from selective reporting of endpoints in this era of public access to protocols [12–15]. A recent study showed that the occurrence of selective outcome reporting of cancer RCTs has decreased substantially since the requirement of public access to protocols, but it is limited by the small sample size of 60 phase III cancer trials reported in a period of 8 months [16].

In the present study, we determined the availability of protocols for phase III cancer RCTs reported recently (2013–2015) in leading journals after the policy of public disclosure of protocols has been endorsed. For trials with publicly accessible trial protocols, we compared primary endpoints reported in publications with those specified in protocols to determine the prevalence of selective reporting of the primary endpoint. We also compared primary endpoints provided in trial registries with those specified in protocols.

## RESULTS

### Availability of trial protocols

Of the 1422 articles retrieved, 343 phase III trials were identified, and protocols were available for 217 of them. The 126 trials without protocols (37%) were published in journals that, at the time of publication, had a stated policy that the protocol should be made available to the public. For these trials there were no links to protocols in reports of 117 trials and 9 protocols could not be found due to non-accessible or expired links. The remaining 217 trials (including two trials with non-English protocols) with publically available protocols consisted of 187 trials with online appended protocols, 4 trials with previously published protocols and 26 trials with protocols available through links included in the text. A full list of the 217 trials is provided in Supplementary Table 2.

The overall 63% availability rate of trial protocols varied among different journals. Nearly all trials (98%) published in NEJM were provided with appended protocols, followed by JCO (88.9%) and JAMA (55.6%). For Lancet and Lancet Oncology, availability rates were 33.0% and 23.5%, respectively. Both univariate and multivariate logistic regression showed that Lancet, Lancet oncology and JAMA were associated with lower availability compared with NEJM (Table 1). After adjusting for other factors, there was a trend of higher availability of protocols over time. Funding source and trials results were not associated with availability of trial protocols in the multivariate regression model. (Table 1)

**Table 1:** Univariate and multivariate logistic regression for factors associated with availability of trial protocols

Variable	Trials with publically available protocols	Trials without publically available protocols	*P* Value	
	No. (%)	No. (%)	Univariate	Multivariate
Year of publication			0.223	0.020
2013	67 (57.3)	50 (42.7)		
2014	74 (67.9)	45 (32.1)		
2015	76 (65.0)	41 (35.0)		
Journal			< 0.001	< 0.001
*The New England Journal of Medicine*	50 (98.0)	1 (2.0)		
*The Lancet*	6 (30.0)	14 (70.0)		
*The Lancet Oncology*	28 (23.5)	91 (76.5)		
*JAMA*	5 (55.6)	4 (44.4)		
*Journal of Clinical Oncology*	128 (88.9)	16 (11.1)		
Trial met primary outcome			0.513	0.842
Yes	104 (61.5)	65 (38.5)		
No	113 (64.9)	61 (35.1)		
Industry funding^†^			0.010	0.138
Yes	136 (58.6)	96 (41.4)		
No	81 (73.0)	30 (27.0)		

^*^Percentages may not total 100 because of rounding.

^†^Including trials fully or partially funded by industry.

### Trial characteristic

**Figure 1:**
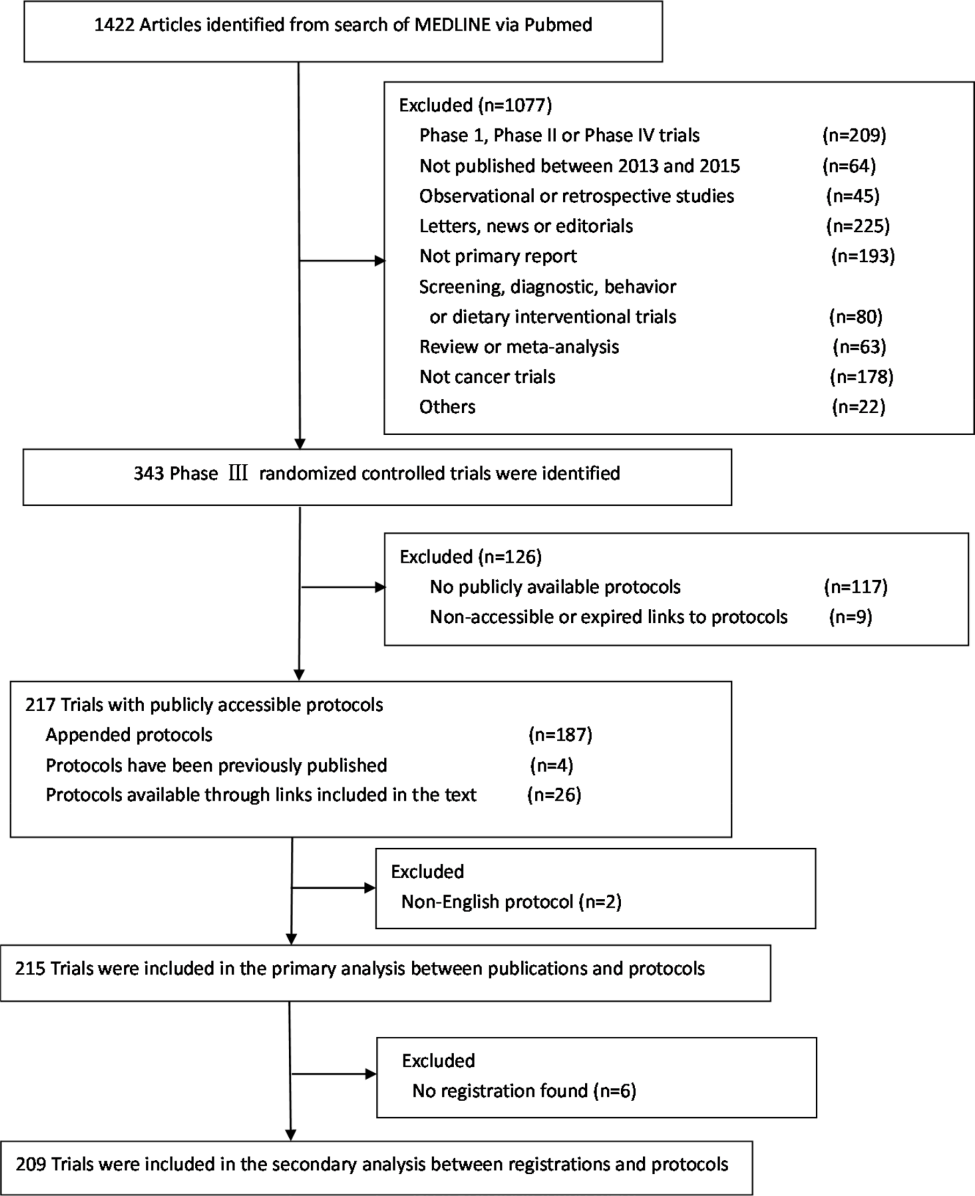
Flowchart of screening of phase III randomized controlled trials included in the study

Phase III RCTs with available protocols in English were included in the primary analysis of comparison of primary endpoints between publications and protocols (Figure 1).Characteristics of these 215 trials are presented in Table 2. The median sample size was 508 (range, 46–7576). Hematologic cancer (16.3%) and breast cancer (15.3%) were the leading cancer types studied. The majority of trials were published in JCO (59.5%); most were at least partially funded by industry (62.3%) and had superiority design (87.4%).

**Table 2:** Characteristics of trials included in the primary analysis between publications and protocols^*^

Characteristic	No. (%) of Trials (*N* = 215)
Type of tumor	
Breast	33 (15.3)
Hematologic	35 (16.3)
Lung	24 (11.2)
Colorectal	15 (7.0)
Other	108 (50.2)
Journal	
*The New England Journal of Medicine*	50 (23.3)
*The Lancet*	6 (2.8)
*The Lancet Oncology*	26 (12.1)
*JAMA*	5 (2.3)
*Journal of Clinical Oncology*	128 (59.5)
Year of publication	
2013	67 (31.2)
2014	73 (34.0)
2015	75 (34.9)
Type of intervention	
Chemotherapy	54 (25.1)
Targeted therapy	102 (47.4)
Hormone therapy	5 (2.3)
Radiation and chemotherapy	11 (5.1)
Surgery or radiation therapy	20 (9.3)
Supportive care	23 (10.7)
Trial met primary endpoint	
Yes	102 (47.4)
No	113 (52.6)
Industry funding	
Yes^†^	134 (62.3)
No	81 (37.7)
Statistical design	
Superiority	188 (87.4)
Equivalence or noninferiority	27 (12.6)
Registry site	
NCT	194 (90.2)
ISRCTN	12 (5.6)
Other or no registration found	9 (4.2)
Sample size	
Median	508
Range	46–7576

Abbreviations: NCT, ClinicalTrials.gov;

ISRCTN, International Standard Randomized Controlled Trial Number Register.

^*^Percentages may not total 100 because of rounding.

^†^Including trials fully or partially funded by industry.

### Analysis between publications and protocols

Nineteen (8.8%) trials reported 2 or more primary endpoints, with a maximum of 3. Overall survival was the primary endpoint in 78 (36.3%) trials, while 111 (51.6%) of trials used another primary time-to-event endpoint. For eight of the 215 trials (3.7%), there was a discrepancy between the primary endpoint defined in the protocol and that reported in the publication (Table 3). The discrepancies consisted of a protocol-defined primary endpoint reported as a non-primary endpoint in the publication (six trials, 2.8%), a protocol-defined primary endpoint omitted in the publication (1 trial, 0.5%), a protocol-defined non-primary endpoint reported as primary endpoint in the publication (2 trials, 0.9%). In three trials (1.4%), different terms were used for the primary endpoint in the protocol and publication, but the definition of the endpoints was similar and thus was not considered to be discrepant. Details of discrepancies and coding are provided in Supplementary Table 3.

**Table 3:** Reporting of primary endpoints in trial publications and discrepancies of primary endpoints between publications and protocols

Variable	No. (%) of Trials (*N* = 2 15)
No. of primary endpoints in trial publications	
Single	196 (91.2)
Multiple	19 (8.8)
Range	1–3
Type of primary endpoints in trial publications^*^	
Overall survival	78 (36.3)
Time to event (excluding overall survival)	111 (51.6)
Response rate	10 (4.7)
Toxicity or symptom scale	23 (10.7)
Other	13 (6.0)
Trials with discrepancies of primary endpoints between publications and protocols^†^	8 (3.7)
Protocol-defined primary endpoint reported as non-primary endpoints in publication	6 (2.8)
Protocol-defined primary endpoint omitted in publication	1 (0.5)
Protocol-defined non-primary endpoint reported as primary endpoints in publication	2 (0.9)
Different terminology of primary endpoints^‡^	3 (1.4)

^*^Some studies had multiple types of primary endpoints

^†^One study has two type of discrepancies

^‡^Two different terms were used for the primary endpoint in the protocol and the publication, but the definition of the endpoints was similar (eg, failure-free survival in protocol, progression-free survival in publication).

**Figure 2:**
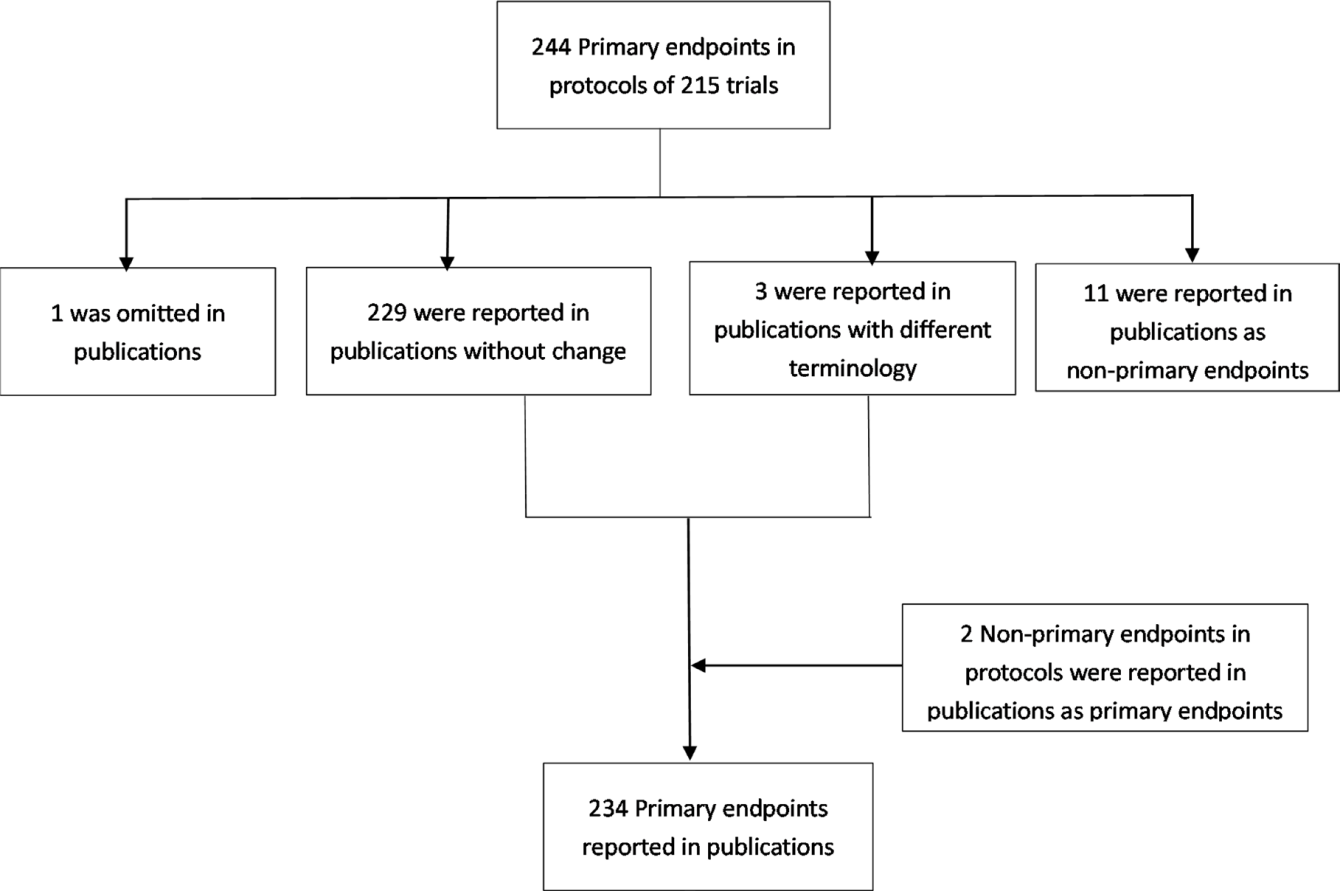
Number of primary endpoints in protocols and publications for 215 trials included in the primary analysis

A total of 244 primary endpoints were identified in protocols of 215 trials. Of these 244 primary endpoints, one was omitted in the publications, 11 were reported as non-primary endpoints, three were reported in publications with different terminology, while 229 (93.9%) were reported without change. A total of 234 primary endpoints were reported in 215 publications. Two were originally designated as non-primary endpoints in the protocols but reported as primary endpoints in publications (Figure 2).

### Analysis between registrations and protocols

Six trials were excluded because no register numbers were found. Two additional trials with non-English protocols were also excluded in this analysis. Discrepancies between the primary endpoints defined in the protocols and registries were identified in 17 of 209 trials (8.1%) (Supplementary Table 4). The protocol-defined primary endpoint was registered as a non-primary endpoint in 2 trials (1.0%); the protocol-defined primary endpoint was omitted in the registry in 9 trials (4.3%); a protocol-defined non-primary endpoint was registered as the primary endpoint in 9 trials (4.3%). In one trial (0.5%), different terms were used for the primary endpoint in the registry and protocol. Details of discrepancies and coding are provided in Supplementary Table 5.

Of 236 primary endpoints identified in 209 protocols, 2 were registered as non-primary endpoints, 9 were omitted in the registrations, one was registered with different terminology, and 224 (94.9%) were registered without change. A total of 241 primary endpoints were registered for 209 trials, including 16 originally protocol-defined non-primary endpoints registered as primary endpoints (Supplementary Figure 1).

## DISCUSSION

Trial outcomes are fundamental to study design and interpretation of results. A main goal of the policy of requiring the public disclosure of protocols for published RCTs is to improve the reliability and consistency of outcome reporting. It allows access to full study details and enhances the credibility of the research [9, 10, 17, 18].

The overall availability of trials protocols to the public for phase III cancer trials published in five leading journals is only 63.3%, despite a policy that protocols of published phase III RCTs should be provided to the public as a condition of publication of reports of phase III RCTs. This indicates that the requirement of providing protocols was not rigorously enforced by journal editors. There is a trend for improvement in availability of trial protocols over time, indicating increasing enforcement by journal editors over this period, although we only analyzed data in recent three years. The availability rates varied among different journals, indicating that stringency in requiring this condition to be met differed in these journals. Different ways of providing protocols may contribute to the different availability rates between these journals. Protocols were provided as online supplements on the websites of NEJM (availability 98%) and JCO (availability 88.9%). For Lancet (availability 33.0%) and Lancet Oncology (availability 23.5%), many links to the trial protocols published on the authors’ institutional websites were not found or not functional.

We identified 215 phase III RCTs with publically available protocols in English. Only 8 of the 215 trials (3.7%) had some discrepancies between the primary endpoint defined in the protocol and that reported in the publication, indicating there has been a clear improvement. Because of the low incidence of discrepancies, a further analysis for the impact of selective outcome reporting is not feasible. Previous studies have shown evidence of selective outcome reporting when primary endpoints were compared between publications and protocols. Chan et al. reported a difference in primary endpoint between trial protocols and publications for 40% of trials in a cohort of trials funded by the Canadian Institutes of Health Research [1]. They also identified discrepancies of primary endpoint in 62% trials using protocols and publication of RCTs approved by a single ethics committee in Denmark [2]. After the policy of registration of trials was embraced by the research community, Mathieu et al. found that 31% of trials showed some evidence of discrepancies between primary endpoints registered and that published [3]. Most of these assessments were conducted before the public disclosure of protocols. More recently, Raghav et al. compared publically available protocols and publications of cancer RCTs, and found that 6.8% of them (excluding 5.4% trials with different terminology of primary endpoint) had some discrepancies between protocol-specified primary endpoint and that reported [16]. However, this analysis was limited by small sample size of 60 phase III trials published in a period of 8 months in 2012. Moreover, phase III trials published in Lancet Oncology have increased substantially and become one of the major resources in oncology community [19], but they were not included in this previous analysis [16]. Thus our study using publically accessible protocols of cancer phase III RCTs published in five major journals in the last three years may better represent the current prevalence of selective outcome reporting. Since the public access to trial protocols became available, discrepancies in primary endpoint in individual trials have been identified by readers and published as letters to these journals [12–15]. This illustrates the substantial impact of public accessibility to trial protocols and probably contributes to the encouraging improvements in selective outcome reporting.

The persistence of small fraction of trials with selective outcome reporting in the era of public access to protocols indicates that the disclosure of protocols to the public needs careful implementation and full involvement of authors, editors, reviewers and readers [10, 11]. Also, lengthy and low quality protocols may present major obstacles for their review during the peer-review process. In the present study, considerable ambiguity of definitions and terminology for the primary endpoint were also found. All these factors increase the difficulties of determining selective outcome reporting by editors, peer reviewers and readers of the publications.

Improvements to protocol quality and peer-review process are needed. Recently, the standardized guidelines for drafting protocols (eg, the Standard Protocol Items: Recommendations for Interventional Trials [SPIRIT] statement [20]) and defining endpoints (eg, the Standardized Definitions for Efficacy Endpoints [STEEP] proposal [21].) have been developed. They demand clear specification of all components of the primary endpoint in the protocol. Increased scrutiny by peer reviewers of reporting of the primary endpoint using the trial protocol as the reference has also been advocated [17]. These efforts may lead to a further decrease in the incidence of selective outcome reporting.

We also found that registered primary endpoint was identical to that in the protocol in only 92.3% of trials. The substantial difference between these two resources would discount the validity of trial registration as a surrogate of protocol. Recently, readers of several studies questioned apparent discrepancies between trial registration entries and corresponding publications. The investigators confirmed that registry entries did not accurately reflect the protocols [12, 13]. Our study has extended this observation and systematically compared primary endpoint in trial registration entries with those in corresponding protocols from public domain. The underlying reasons for this inconsistency are unknown. However, if the information in registries is not faithful to protocols, the previously observed protocol deviation may in fact result from vague, erroneous, or out-of-date registry entries [12]. It has also been suggested that in the practice of registration, registration of trials may be seen as administrative rather than scientific and is frequently delegated to junior team members, leading to the omission of primary endpoint or failure to distinguish the primary and non-primary endpoints [13, 22]. Faced with such a situation, improved registry practices had been proposed recently. Specifically, registration of the full study protocol and amendments was recommended for this purpose [12, 13].

This study has limitations. First, we selected only trials published in five top journals. They may not reflect reports of trials published elsewhere. However, many practice-changing cancer phase III RCTs are published in these journals and they are among the earliest journals that embraced the policy of disclosure of protocols to the public. Moreover, only 62.9% of protocols of phase III cancer trials from these journals were available for further analysis. Thus, we probably provide a lower estimate of the prevalence of selective reporting of outcomes. Second, we did not address the problem of selective reporting of non-primary endpoints, which may be more common.

In conclusion, despite the current policy of unrestricted public access to protocols of RCTs in top journals, provision of protocols remains incomplete. Discrepant reporting of primary endpoints has improved substantially and only remains in a small fraction of phase III trials. Further improvement in consistency between the primary endpoint registered and that in protocol is necessary.

## MATERIALS AND METHODS

### Articles selection

We searched MEDLINE via PubMed to identify phase III cancer RCTs published between January 2013 and December 2015 in “New England Journal of Medicine (NEJM), Lancet, Lancet Oncology, JAMA and Journal of Clinical Oncology (JCO)”*.* We selected articles published in these journals because they all have a high impact factor > 20 and also initiated a policy between 2009 and 2011 that protocols should be provided to the public as a condition of acceptance (specific trial protocol policies of these journals are provided in Supplementary Table 1). The cochrane Highly Sensitive Search Strategy for identifying RCTs was used. Details of the search strategy are provided in the Supplementary. A hand search of the journals’ tables of contents was conducted to supplement previous search results.

We screened title, abstract or full text of each article to identify phase III trials. We excluded letters, news or editorials, reviews and meta-analysis, observational or retrospective studies, non-phase III studies, or non-primary reports, non-cancer trials, screening, diagnostic, behavior, or dietary trials.

For each publication of a clinical trial, we searched systematically to obtain the protocol of the trial: published as an online supplement, published previously and referenced by the authors, or via a link to the protocol in the publication.

We determined whether the protocol was available for each trial and calculated the proportion of available protocols.

We identified the registry entry of each trial by its register number provided online or included in the text, or by searching ClinicalTrials.gov and the International Standard Randomized Controlled Trial Number Register.

### Data collection

For trials with available English language protocols, we compared the primary endpoint defined in the protocol with that reported in the publication. If multiple versions of the protocol were provided, the most recent was used for data collection. To determine the primary endpoints from the protocol, preference was given to section in this order: primary endpoints, primary objectives, endpoint stated in the sample size calculation [2, 3].

Primary endpoints from protocols, publications, and registrations were extracted into a spreadsheet and compared in publications/protocols and registrations/protocols because information in the protocol should be the truth. We classified discrepancies between the primary endpoints described in the protocols and those specified in the publications (registrations) into four categories according to a classification modified from previous studies [1–3]: protocol-defined primary endpoint reported as non-primary endpoints in the publication; protocol-defined primary endpoint omitted in the publication; protocol-defined non-primary endpoint reported as primary endpoint in the publication; new primary endpoint not defined in protocol was introduced in the publication. If the terminology of the primary endpoint was changed (e.g., progression-free survival and time to progression), detailed definition of the terminology was compared. Trials could be assigned more than one type of discrepancy. If the authors of the publication noted the change of primary endpoint between protocol and publication and provided reasonable explanation, we did not consider this a discrepancy. Because the time-frame for time-to-event endpoints was often omitted in the definition of the primary endpoint, and predefined time limits in oncology trials were frequently revised due to decreased or increased event rates or interim safety or efficacy analysis, we did not consider a change of timing of assessment of the primary endpoint as a discrepancy.

Additional data were also extracted: cancer types, journal, year of publication, intervention type, whether the primary endpoint was met, funding source, statistical design, registry website, and sample size.

Two authors (S.Z and F.L.) independently extracted relevant data. Disagreement was resolved by consensus of all authors through reappraisal of original documents. We used the κ coefficient to determine the degree of agreement between reviewers. Agreement between reviewers was high (κ = 0.92 for trial selection and data abstraction, 0.89 for determination and classification of discrepancies).

### Statistical analysis

The primary objectives of this study were to determine (i) the proportion of published reports of phase III cancer RCTs that had a publicly-available protocol, and factors associated with availability of trial protocols; (ii) the proportion of trials with a discrepancy in primary endpoints between their published reports and corresponding protocols. The secondary objective was to evaluate consistency of primary endpoints between trial registries and protocols.

Categorical variables are reported as frequencies and percentages. Continuous variables are described as medians and range. Univariate and Multivariate Logistic Regression were used to explore factors associated with availability of trial protocols. Factors explored included year of publication, journal, funding source and trial results. Factors were considered statistically associated with available protocols if the associated *P* < 0.05. Data analysis was performed using SPSS 16.0 (SPSS, Chicago, IL, USA).

## SUPPLEMENTARY MATERIALS FIGURE AND TABLES




