# Supplementary material for: Comparison of primary endpoints between publications, registries, and protocols of phase III cancer clinical trials
Source: Oncotarget. 2017 Oct 3;8(57):97648–56. doi: 10.18632/oncotarget.21459 (PMC5722592; doi:10.18632/oncotarget.21459)
Supplement: Supplementary file 1 [file oncotarget-08-97648-s001.pdf]

Comparison of primary endpoints between publications, registries, and protocols of phase III cancer clinical trials

SUPPLEMENTARY MATERIALS

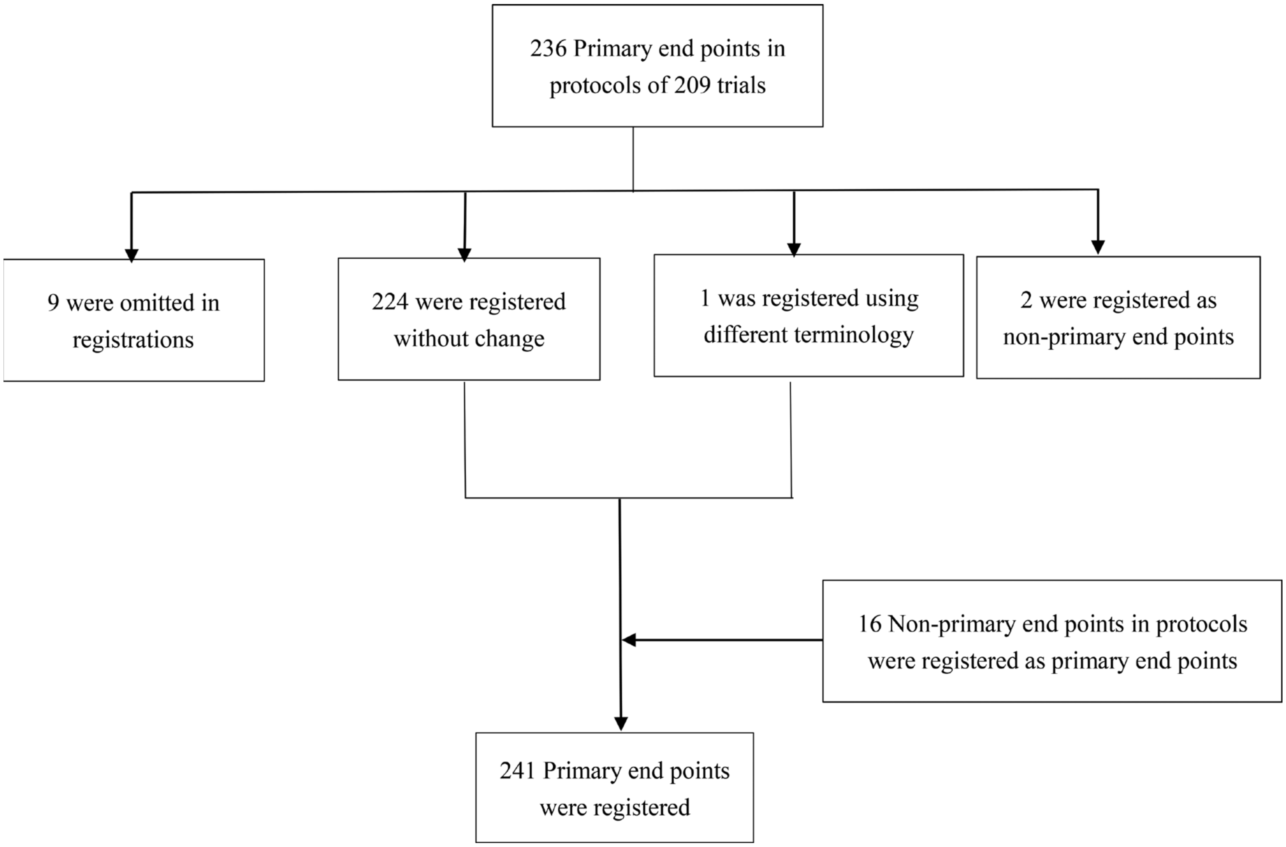

Supplementary Figure 1: Number of primary end points in protocols and registrations for 209 trials included in the secondary analysis.

Supplementary Table 1: Specific trial protocol policies of the 5 journals reviewed

Journal	Instruction for authors	Related editorials or comments
The New England Journal of Medicine	The protocols of a clinical trial should be submitted as a separate PDF file, independent of the Supplementary Appendix. A statistical analysis plan may be included with the protocol, in the same PDF document.[1]	
The Lancet	Reports of trials must conform to CONSORT 2010 guidelines, and should be submitted with their protocols.<br>All accepted Articles should include a link to the full study protocol published on the authors' institutional website.[2]	To help authors demonstrate that their findings are faithful to their research protocol, The Lancet, The Lancet Oncology, and The Lancet Neurology offer to publish links to the full study protocol on the authors' institutional website.[3]
The Lancet Oncology	Reports of trials must conform to CONSORT 2010 guidelines, and should be submitted with a full protocol in English, or, with the full protocol and a synopsis in English including details of enrolment criteria, outcomes/endpoints, and statistical considerations.<br>All accepted Articles should include a link to the full study protocol published on the authors' institutional website[4]	To help authors demonstrate that their findings are faithful to their research protocol, The Lancet, The Lancet Oncology, and The Lancet Neurology offer to publish links to the full study protocol on the authors' institutional website.[3]
JAMA	Authors of manuscripts reporting clinical trials must submit trial protocols (including the complete statistical analysis plan) along with their manuscripts. If the manuscript is accepted, the protocol will be published as an online supplement.[5]	
Journal of Clinical Oncology	JCO believes that for the editors and reviewers to properly peer review a submission, as well as for readers to thoroughly interpret an article, a redaction of the protocol for all randomized phase II and III studies must be provided. This applies to reports of primary planned endpoints of the study, as well as planned or unplanned subset analyses. It is the responsibility of the authors to submit only the following sections (although submission of the full protocol would be acceptable as well). The information provided must reflect the most recent, revised version of the protocol.<br><br>Selection of patients, including both eligibility and ineligibility criteria<br>Schema and treatment plan, including administration schedule<br>Rules for dose modification<br>Measurement of treatment effect including response criteria, definitions of response and survival, and methods of measurement<br>Reasons for early cessation of trial therapy<br>Objectives and entire statistical section (including endpoints)<br>Authors should upload this material as a Supplemental File. It will be available to the editors and reviewers during the peer review process and, if your manuscript is accepted, will be published online.[6]	For randomized phase II and III trials, JCO will now permit readers access to the following redacted portions of the most recent protocol: selection of patients, including both eligibility and ineligibility criteria; schema and treatment plan, including administration schedule; rules for dose modification; measurement of treatment effect, including response criteria, definitions of response and survival, and methods of measurement; reasons for early cessation of trial therapy; and objectives and the entire statistical section, including endpoints.[7]

1 The New England Journal of Medicine Author center: Supplementary Appendix. http://www.nejm.org/page/author-center/supplementary-appendix
2 The Lancet: Information for Authors. http://www.thelancet.com/lancet/information-for-authors
3 Summerskill W , Collingridge D, Frankish H. Protocols, probity, and publication. Lancet 2009; 373: 992.
4 The Lancet Oncology: Information for Authors. http://www.thelancet.com/lanonc/information-for-authors
5 JAMA: Instructions For Authors. http://jama.jamanetwork.com/public/instructionsForAuthors.aspx
6 Journal of Clinical Oncology: Author Center | How Do I Submit My Manuscript? http://jco.ascopubs.org/site/ifc/submit-my-manuscript.xhtml#randomized-studies
7 Haller DG, Cannistra SA: Providing protocol information for Journal of Clinical Oncology readers: What practicing clinicians need to know. J Clin Oncol 29:1091, 2011

Supplementary Table 2: List of Phase III trials with publicly accessible protocol. See Supplementary_ Table_2

Supplementary Table 3: List of discrepancies of primary endpoints between protocols and publications (including different terminology of primary endpoint)

Trial	Protocol-defined primary endpoints	Reported primary endpoints	Type of discrepancies*
Trial 1	DFS	RFS	4
Trial 2	EFS	EFS	NA
	OS		1
Trails 3	OS	OS	NA
	Frequency and severity of toxicity		1
Trial 4	Time to progression to symptomatic	Time to progression to symptomatic	NA
	Response rates		1
	Response duration		1
Trial 5	TTP	PFS	2 and 3
Trial 6	OS	OS	NA
	Complete remission		1
	Duration of remission, relapse rates and deaths in first complete remission		1
	Toxicity, both haematological and non-haematological, and quality of life (QoL)		1
	Supportive care requirements		1
Trial 7	OS	OS	NA
	Toxicity		1
Trial 8	FFS	PFS	4
Trial 9	Time to initiation of therapy	Time to start of new treatment	NA
		Quality of life at month 7	3
Trial 10	Local recurrence	Local failure	4
Trial 11	Composite cognition score	Composite cognition score	NA
	Subjective cognitive complaints/ symptoms		1
	Fatigue		1

Abbreviation: DFS, disease-free survival; RFS, relapse-free survival; EFS, event-free survival; OS, overall survival; TTP, time to progression; PFS, progression-free survival; FFS, failure-free survival; NA, not applicable.

*1 denotes protocol-defined primary endpoint reported as non-primary endpoint in publication; 2 denotes protocol-defined primary endpoint omitted in publication; 3 denotes protocol-defined non-primary endpoint reported as primary endpoint in publication; 4 denotes different terminology of primary endpoint.

Supplementary Table 4: Discrepancies of primary endpoints between registrations and protocol

Variable	No.(%) of Trials (*N* = 209)
Trials with discrepancies of primary endpoints between registrations and protocols*	16 (7.7)
Protocol-defined primary endpoint registered as non-primary endpoint	2 (1.0)
Protocol-defined primary endpoint omitted in registrations	9 (4.3)
Protocol-defined non-primary endpoint registered as primary endpoint	9 (4.3)
Different terminology of primary endpoint†	1 (0.5)

*Some studies had multiple types of discrepancies

†Two different terms were used for the primary endpoint in the protocol and the registration, but the definition of the endpoints was similar (eg, failure-free survival in protocol, progression-free survival in registration).

Supplementary Table 5: List of discrepancies of primary endpoints between protocols and registrations (including different terminology of primary endpoint)

Trial	Protocol-defined primary endpoints	Registered primary endpoints	Type of discrepancies*
Trial 1	OS	No primary endpoint provided in registration	2
Trial 2	OS	OS	NA
		PFS	3
		Quality of life	3
		Treatment costs	3
		Toxicity and tolerance	3
		Tumor profiles of gene expression as measured by biochips with DNA and tissue microarrays	3
Trial 3	Pain score measured using a Visual Analogue Score	Pain score measured using a Visual Analogue Score	NA
	Pleurodesis failure		1
Trial 4	OS	OS	NA
		DFS	3
Trial 5	OS	OS	NA
		PFS	3
Trail 6	OS	OS	NA
	Toxicity	Toxicity	NA
		PFS	3
		Tumor Response	3
Trial 7	DFS	DFS	NA
		EFS	3
		OS	3
Trial 8	OS		2
	Quality of life	Quality of life	NA
Trial 9	Time to progression to symptomatic	Time to progression to symptomatic	NA
	Response rates		1
	Response duration		2
Trial 10	Hand-foot syndrome of any grade within a 6-week time	No primary endpoint provided in registration	2
Trial 11	TTP	PFS	2 and 3
Trial 12	OS	No primary endpoint provided in registration	2
Trial 13	FFS	PFS	4
Trial 14	PFS	TTP	2 and 3
Trial 15	OS	No primary endpoint provided in registration	2
Trial 16	DFS	DFS	NA
		OS	3
		Toxicity	3
Trial 17	PFS	TTP	2 and 3

Abbreviation: OS, overall survival; PFS, progression-free survival; DFS, disease-free survival; EFS, event-free survival; TTP, time to progression; FFS, failure-free survival; NA, not applicable.

*1 denotes protocol-defined primary endpoint registered as non-primary endpoint; 2 denotes protocol-defined primary endpoint omitted in registration; 3 denotes protocol-defined non-primary endpoint registered as primary endpoint; 4 denotes different terminology of primary endpoint.
